# Supplementary material for: Explainable AI-based analysis of human pancreas sections identifies traits of type 2 diabetes
Source: Nat Commun. 2026 Feb 9;17:1558. doi: 10.1038/s41467-026-69295-2 (PMC12894717; doi:10.1038/s41467-026-69295-2)
Supplement: Supplementary file 1 — Supplementary Information [file 41467_2026_69295_MOESM1_ESM.pdf]

Supplementary Material

Supplementary Figures

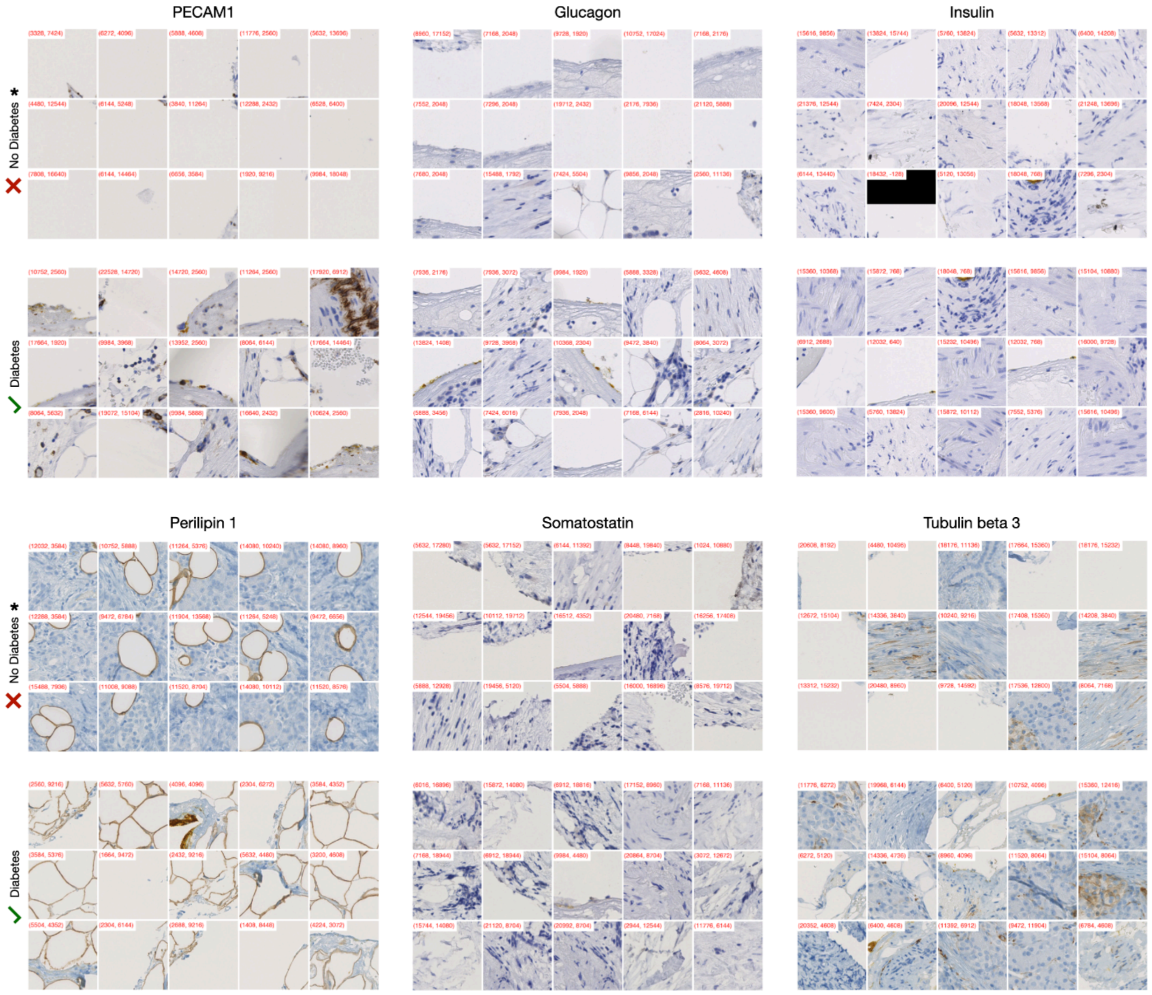

Suppl. Figure 1: Representative top 15 attended patches from each chromogenic IHC staining associated with the outcomes ‘diabetes’ and ‘no diabetes’.

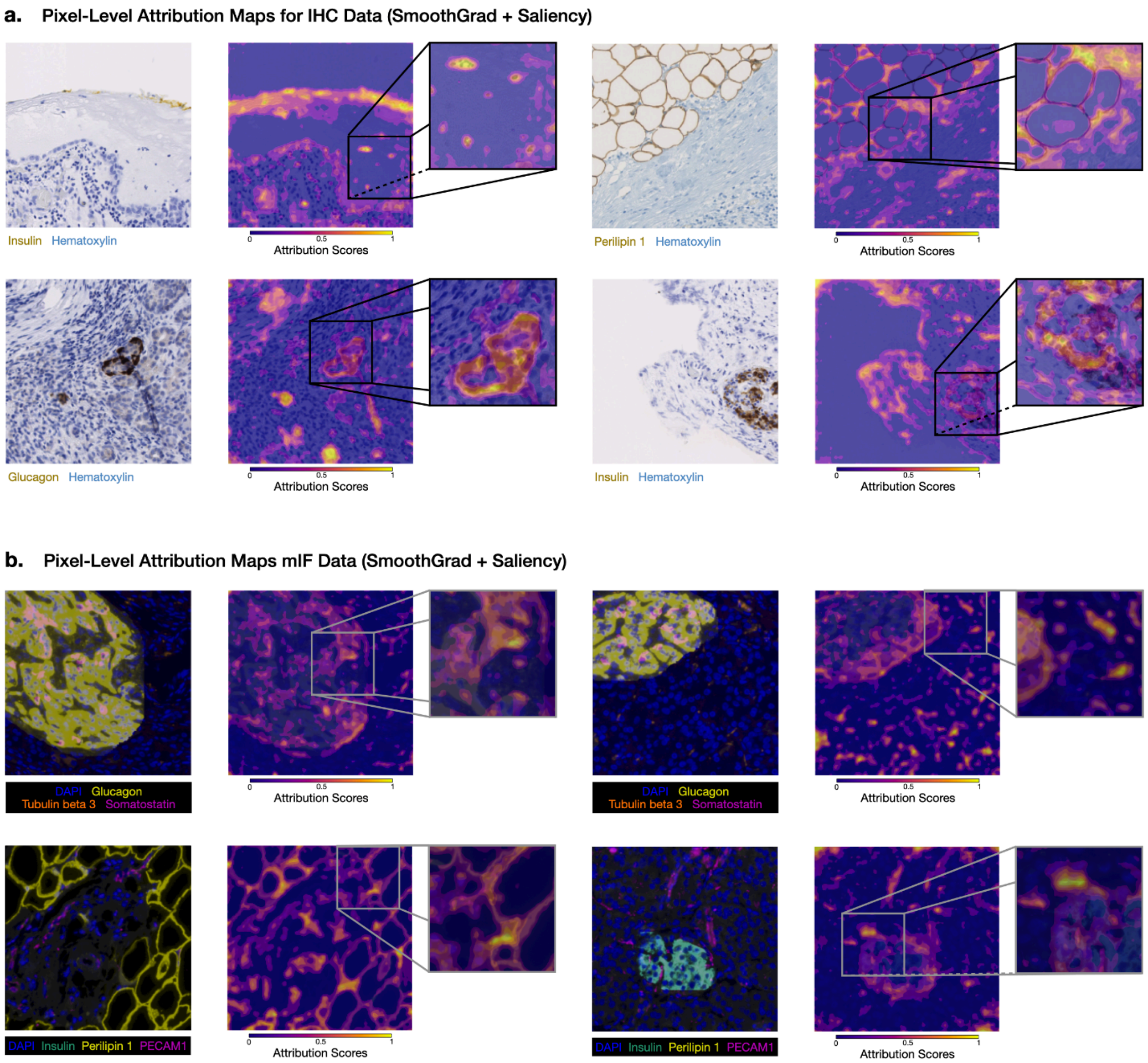

Suppl. Figure 2: Pixel-level heatmaps of local regions and one single patch based on Saliency with SmoothGrad for IHC (a.) and mIF (b.) data.

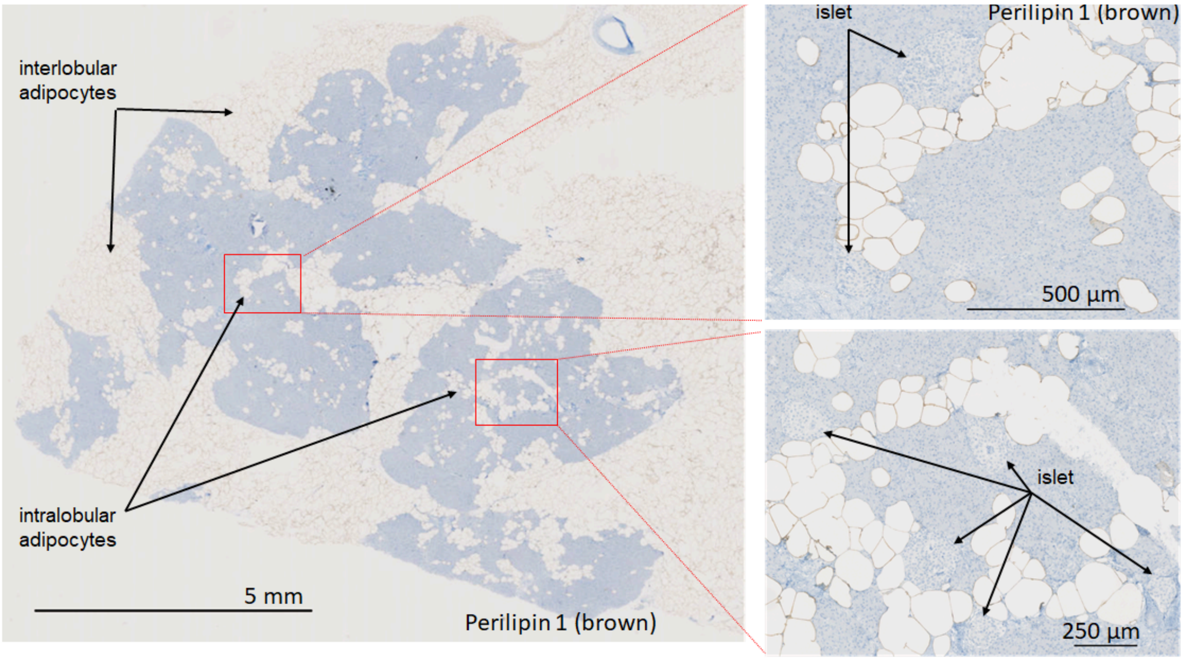

Suppl. Figure 3: Representative IHC image (zoom-in) of a pancreatic section from a non-diabetic patient stained for the adipocyte marker perilipin 1 (brown), showing inter- and intra-lobular infiltration of pancreatic parenchyma with adipocytes. Note the close proximity between adipocytes and some of the islets.

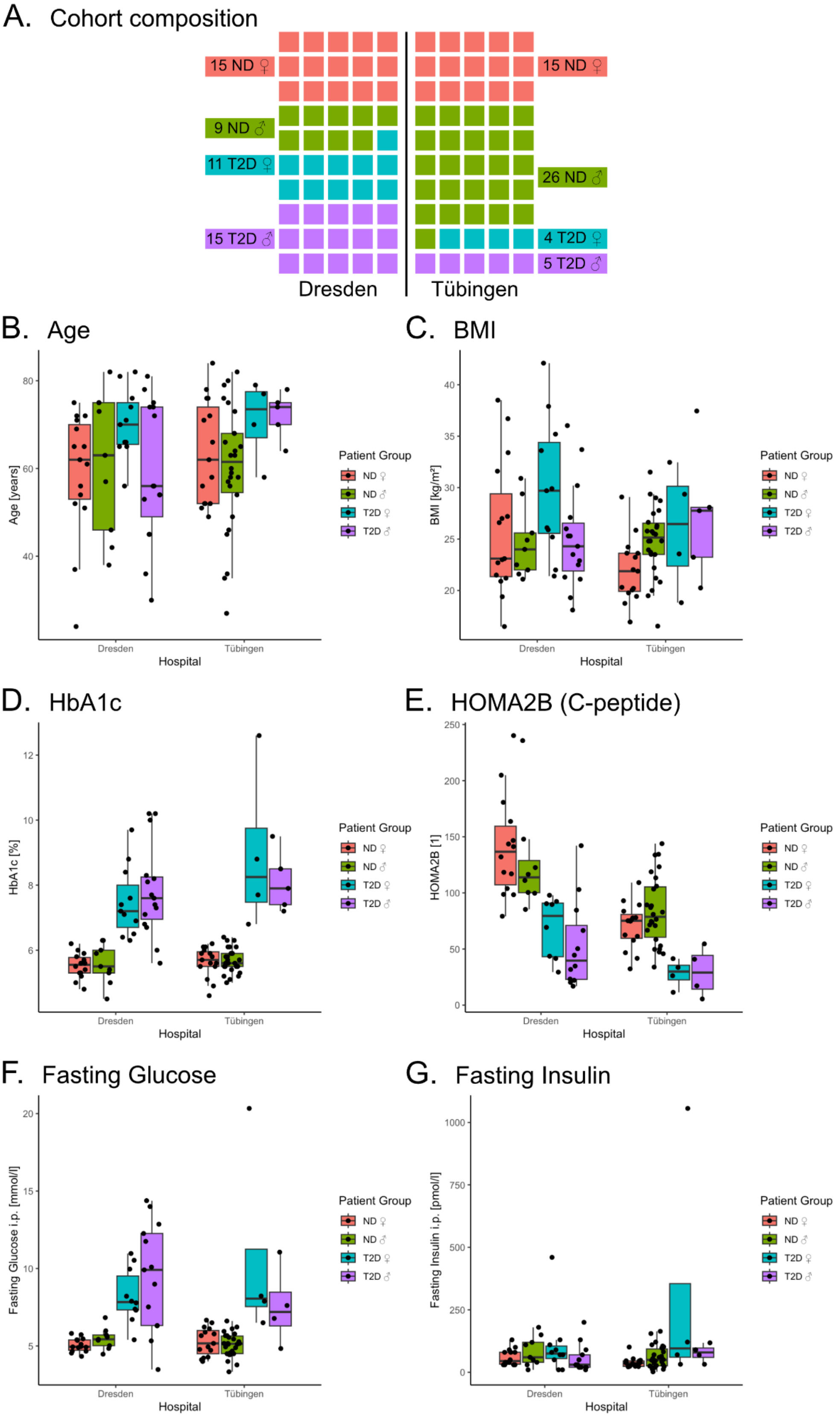

Suppl. Figure 4: Composition and selected clinical data of the analyzed cohort**.** 100 pancreatectomized patients were recruited for this study, including female (♀) and male (♂) individuals with non-diabetic or type 2 diabetic (T2D) background. 50 patients each were

treated due to different indications (e.g. pancreas carcinoma or pancreatitis) at the University Hospitals in Dresden and Tübingen (a.). T2D patients were diagnosed as such according to their clinical records minimum of one year before the onset of the pancreatic indication. All non-diabetic patients do not fulfill the diagnostic criteria for T2D defined by the American Diabetes Association (ADA). Clinical data regarding age (b.), body weight via body mass index (BMI, c.), long-term hyperglycemia via glycated hemoglobin (HbA1c, d.), β-cell function, and insulin resistance via homeostasis model assessment 2B including c-peptide (HOMA2B, e.), fasting glucose (f.), and fasting insulin (g) are shown.

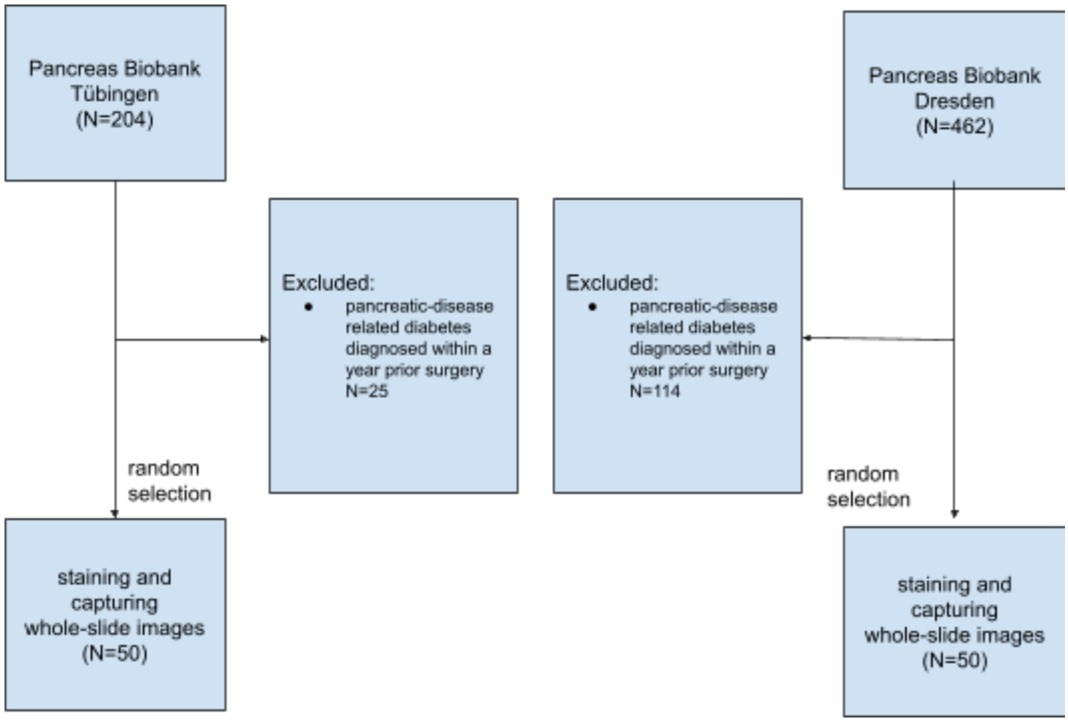

Suppl. Figure 5: Sample selection process.

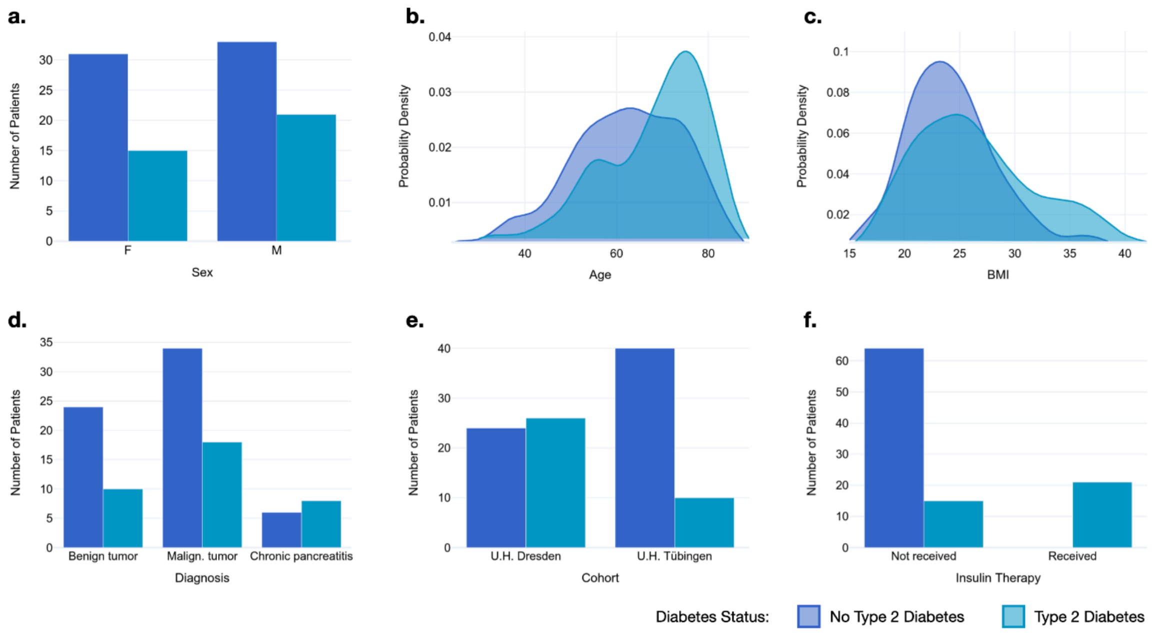

Suppl. Figure 6: Distribution of patient clinical data. Distribution of all control variables between both glycaemic statuses, i.e. no diabetes vs T2D, excluding the immunohistological stainings: sex (a.), age (b.), BMI (c.), condition underlying pancreatic surgery (d.), cohort (e.), and insulin therapy (f.) (n=100).

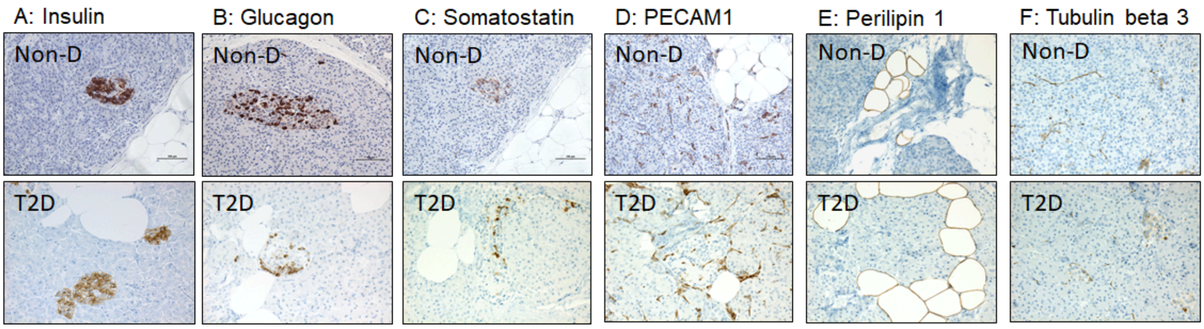

Suppl. Figure 7: Representative brightfield IHC microscopy images (20x magnification) of pancreatic resections from a normal glucose-tolerant patient (A-F: upper panels) and a patient with T2D (A-F: lower panels). Sections of formalin-fixed, paraffin-embedded human pancreatic tissue were stained (in brown) for (A) insulin, (B) glucagon, (C) somatostatin, (D) PECAM1, (E) perilipin 1, (F) tubulin beta 3, and counterstained with hematoxylin (blue) as described in Methods.

(a.) Non-diabetic

Staining set 1:

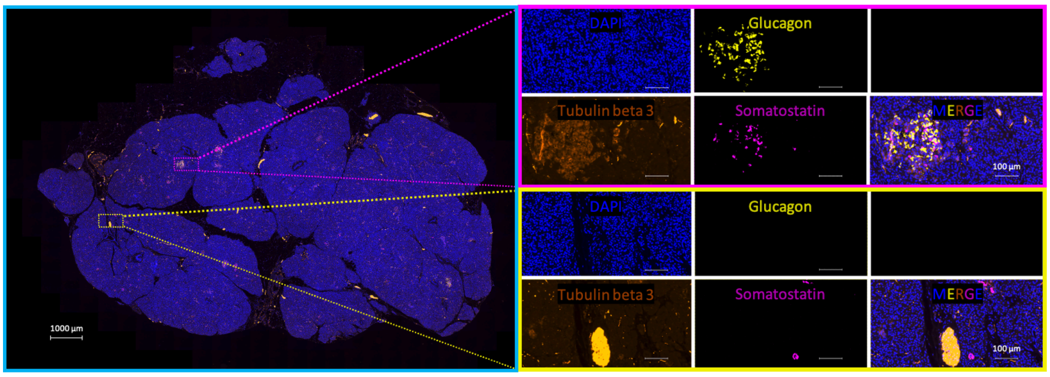

Staining set 2:

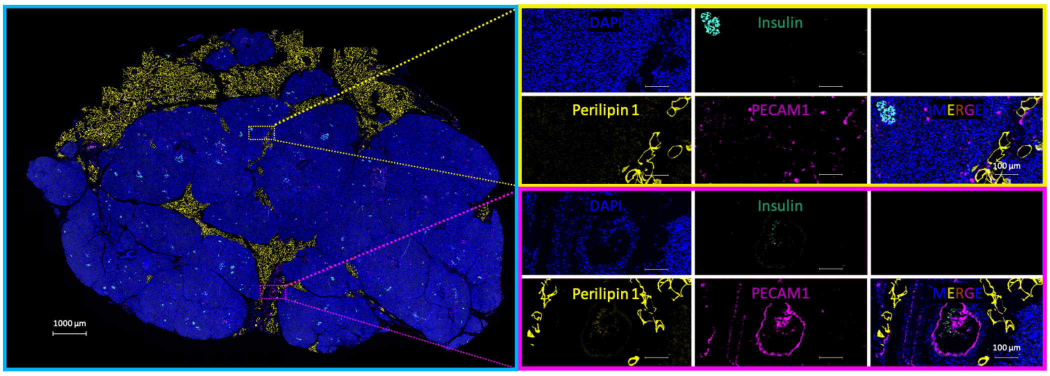

(b.) T2D

Staining set 1:

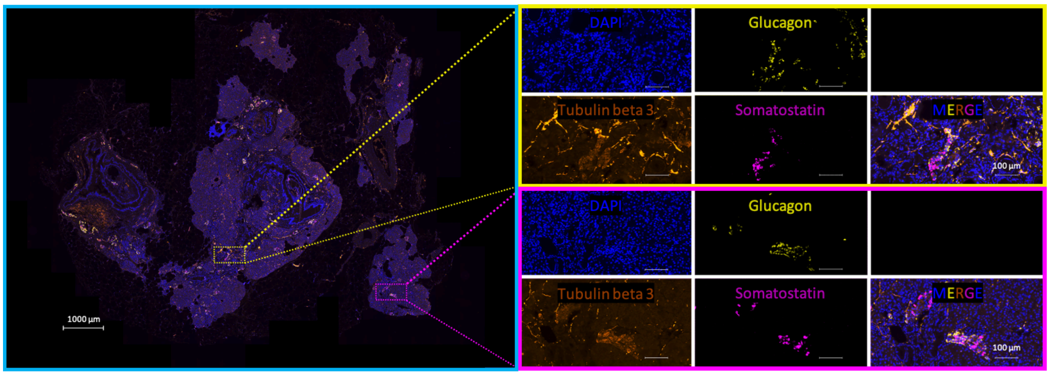

Staining set 2:

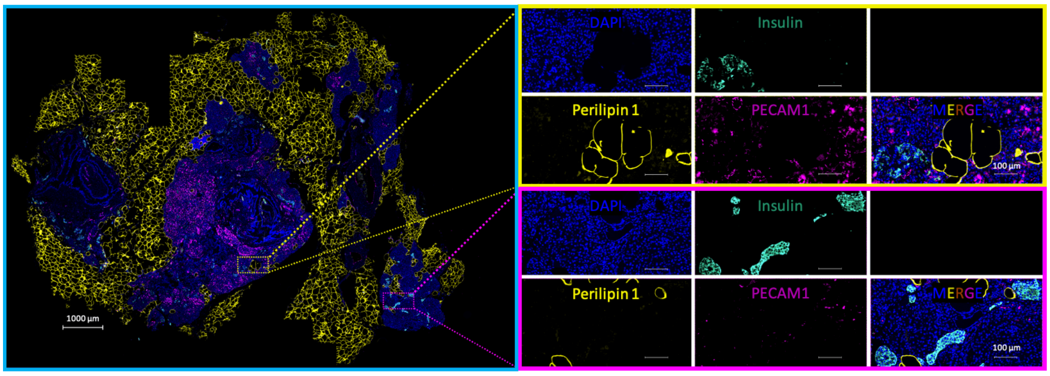

Suppl. Figure 8: Representative mIF images of pancreatic resections from the same normoglycemic patient (a.) and the same patient with type 2 diabetes (b.) as showcased for chromogenic stainings in Suppl. Figure 7. Nuclear DNA is visualized using DAPI. Staining set 1: glucagon and somatostatin are markers for pancreatic α-and δ-cells, respectively whereas tubulin beta 3 marks neuronal axons (intense fluorescence) as well as islet cells (moderate fluorescence). Staining set 2: insulin, perilipin 1, and PECAM1 are markers for pancreatic β-cells, adipocytes, and vascular endothelial cells, respectively. Whole-slide images were captured as tile scans with 20x magnification. Overview images's scale bar: 1000 µm; detail image's scale bar: 100 µm.

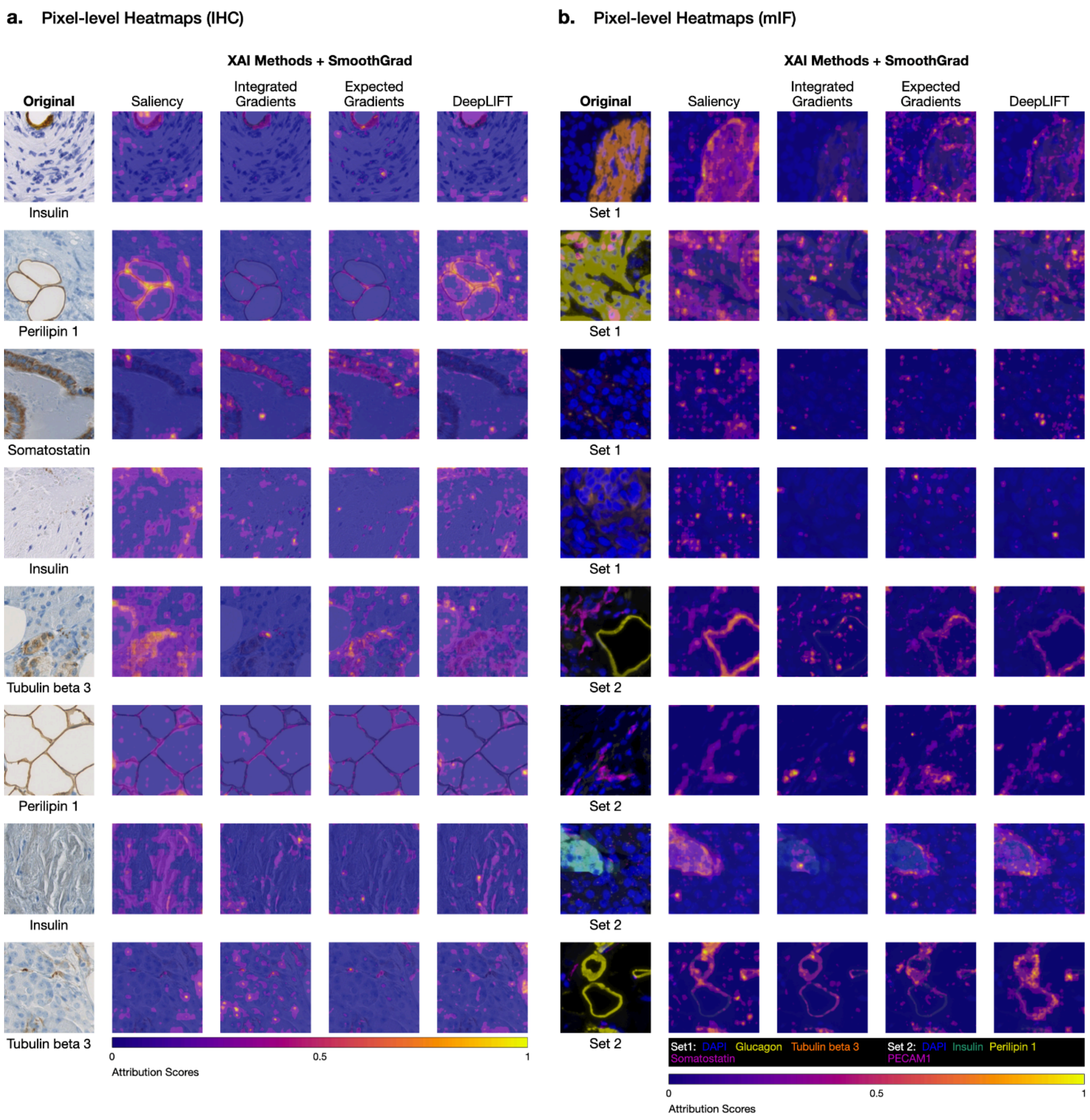

Suppl. Figure 9: Exemplary pixel-level heatmaps of all four applied attribution methods for IHC (a.) and mIF (b.) stained patches. They differ in granularity and in rare occasions also in attributed regions.

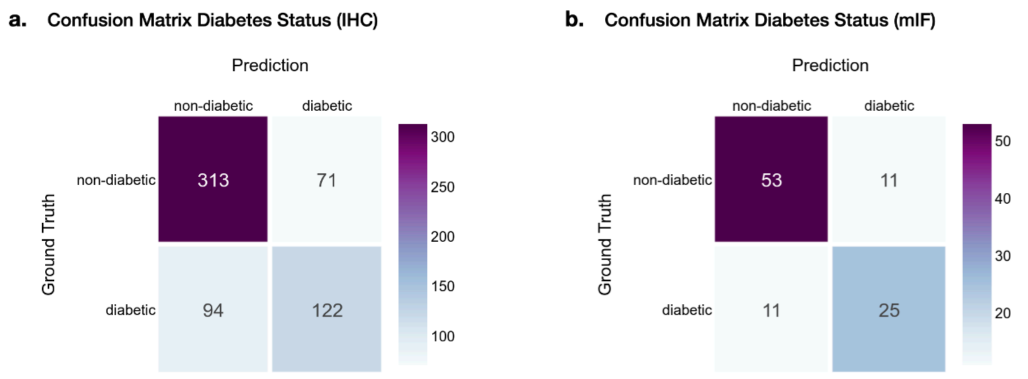

Suppl. Figure 10: Logistic MLM confusion matrices for IHC (n=600) (a.) and mIF (b.) data (n=100).

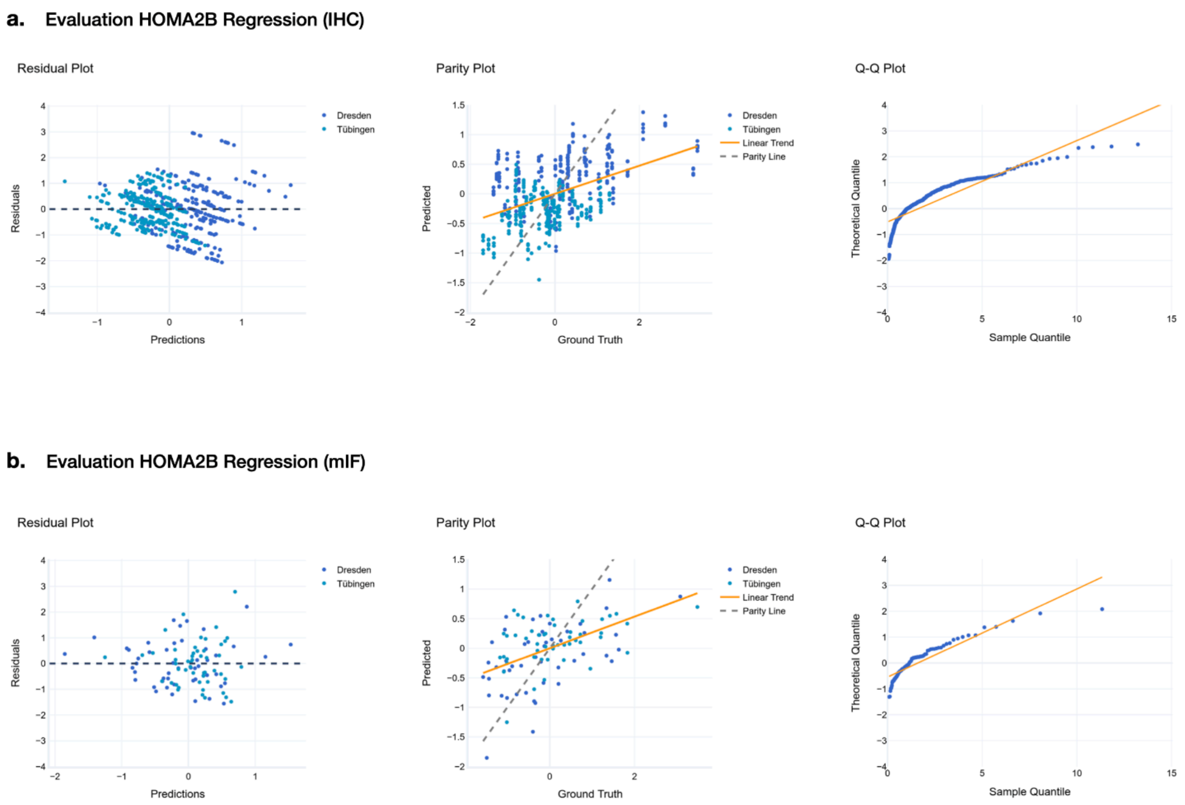

Suppl. Figure 11: Residual, Parity, and Q-Q Plot for HOMA2-B predicting MLMs on IHC (n=600) (a.) and mIF (b.) data (n=100).

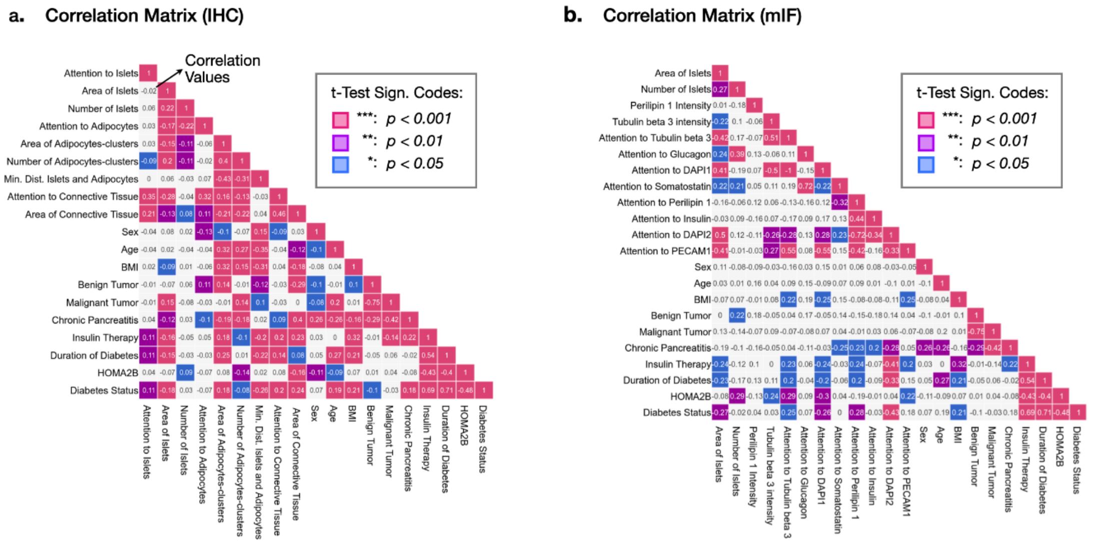

Suppl. Figure 12: Correlation between biomarker and clinical patient data for IHC (a.) and mIF (b.) data sets. Values show the Pearson correlation and colors indicate their significance based on a two-sided two-sample t-Test.

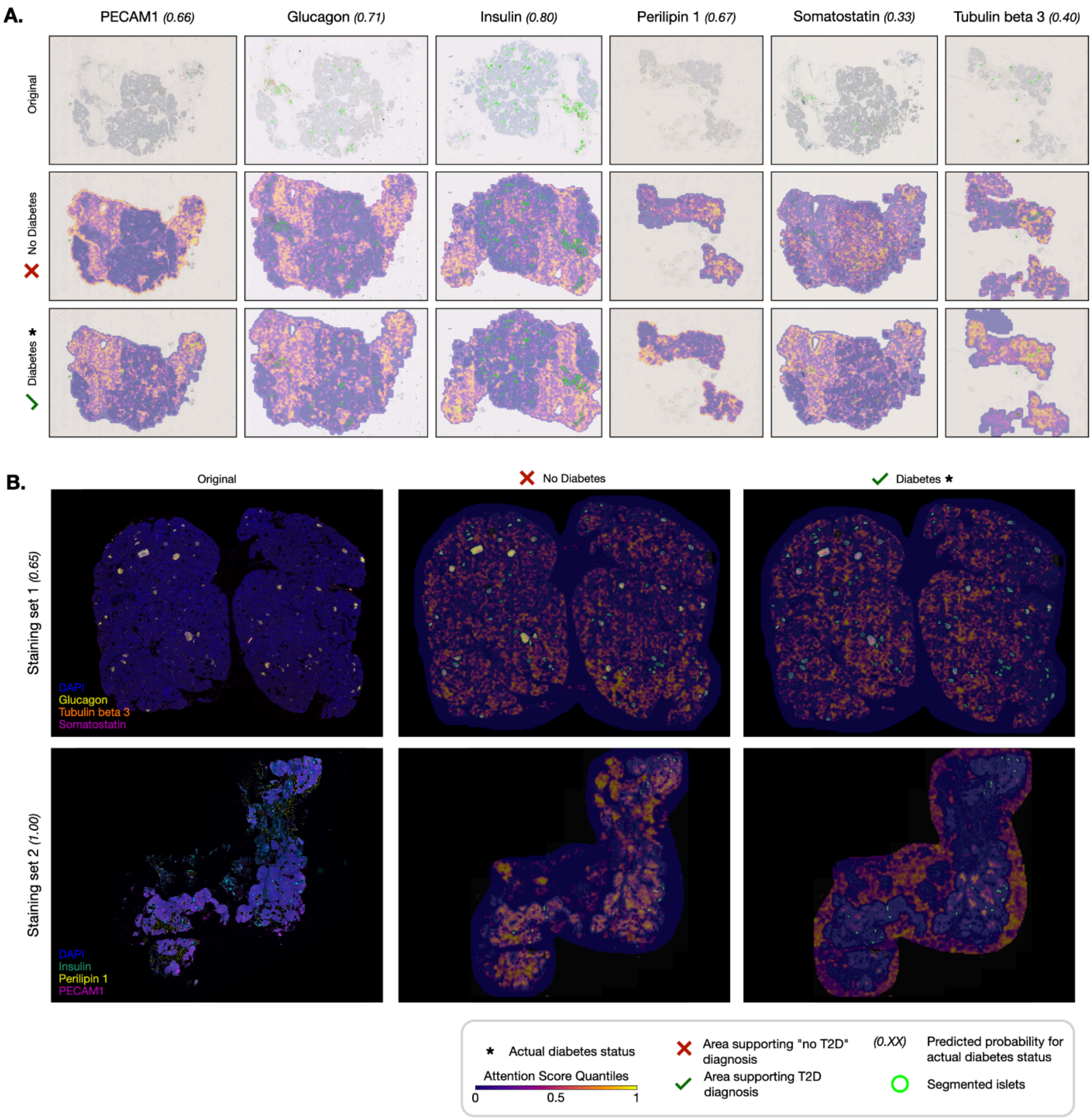

Suppl. Figure 13. Representative attention heatmaps for the IHC and mIF WSIs of a single patient. Attention-based heatmaps on global WSI level for (a.) each IHC staining and (b.) Staining sets 1 and 2 of mIF staining.

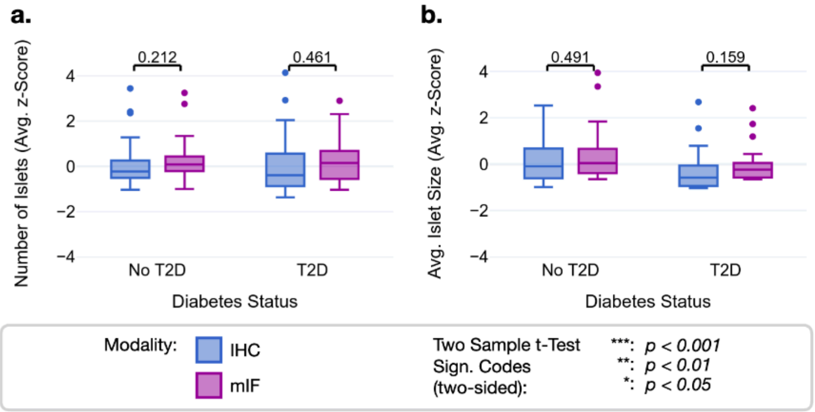

Suppl. Figure 14: Distribution of number of islets and the average islets size computed only from the brightfield or fluorescence WSIs. Respective p-values (two-sided two-sample t-test) indicate significant differences between 100 randomly sampled observations from the IHC and mIF microscopy data. We observe that there is no significant difference. The box plots show the quartiles, with the median (i.e. second quartile) marked by a line inside.

Supplementary Tables

(a.) Ensemble Performance on IHC WSIs

Representation	Stainingset	Encoder / MIL Algorithm	AUROC (higher is better)
Channel-wise Average	1	Imagenet / CLAM	**0.956**
	2	Imagenet / CLAM	0.684
Channel-wise	1	Imagenet / CLAM	**0.912**
	2	Imagenet / CLAM	0.816
RGB	1	Imagenet / CLAM	**0.842**
	2	Imagenet / CLAM	0.640

(b.) Ensemble Performance on mIF WSIs

Staining	MIL Algorithm	Encoder	AUROC (higher is better)
Mean over Stainings	CLAM	Imagenet	**0.833**
	Chowder	Imagenet	0.830
	CLAM	Phikon	0.794
	Chowder	Phikon	0.781
Tubulin beta 3	CLAM	Imagenet	**0.895**
Insulin	CLAM	Imagenet	0.842
Glucagon	CLAM	Imagenet	0.842
Perilipin 1	CLAM	Imagenet	0.842
Somatostatin	CLAM	Imagenet	0.842
PECAM1	CLAM	Imagenet	0.737
Tubulin beta 3	CLAM	Phikon	0.851
Insulin	CLAM	Phikon	0.684
Glucagon	CLAM	Phikon	0.711
Perilipin 1	CLAM	Phikon	0.816
Somatostatin	CLAM	Phikon	**0.921**
PECAM1	CLAM	Phikon	0.781
Tubulin beta 3	Chowder	Imagenet	**0.877**
Insulin	Chowder	Imagenet	0.851
Glucagon	Chowder	Imagenet	0.640
Perilipin 1	Chowder	Imagenet	**0.877**
Somatostatin	Chowder	Imagenet	**0.877**
PECAM1	Chowder	Imagenet	0.851
Tubulin beta 3	Chowder	Phikon	0.833
Insulin	Chowder	Phikon	0.693
Glucagon	Chowder	Phikon	0.728
Perilipin 1	Chowder	Phikon	**0.860**
Somatostatin	Chowder	Phikon	0.842
PECAM1	Chowder	Phikon	0.728

Suppl. Table 1: Ensemble AUROC for prediction performance of each model for the IHC (a.) and mIF (b.) WSIs on the held-out test set consisting of 25 patients.

	Tübingen (% or mean±SD)	Dresden (% or mean±SD)
Sex (females)	38 %	52 %
Age (years)	63.2 ± 12.97	61.8 ± 14.35
BMI (kg/m2)	26.33 ± 5.8	24.30 ± 4.1
Diabetes (%)	18	52
- diabetes duration (years)	2.2 ± 6.5 (0-35 years)	5.7 ± 8.8 (0-30 years)
- metformin treatment	6 (12 %)	11 (22%)
- sulfonylurea treatment	1 (2%)	2 (4%)
- GLP-1 analogon treatment	0 (0 %)	3 (6%)
- SGLT2-inhibitor treatment	0 (0 %)	1 (2 %)
- insulin treatment	6 (12 %)	15 (30%)
Fasting glucose (mmol/l)	5.8 ± 2.49	7.07 ± 2.78
Fasting insulin (pmol/l)	76.80 ± 147.8	74.35 ± 74.08
HOMA-2B (C-peptide)	70.76 ± 32.15	99.58 ± 55.56
HbA1c (%)	6.17 ± 1.35	6.67 ± 1.44
Diagnosis		
- Malignant disease	58 %	46 %
- Chronic Pancreatitis	6 % (3 out of 50)	22 % (11 out of 50)
- Other	36 %	32 %

Suppl. Table 2: Clinical cohort characteristics.

a. GMLM Regression for Diabetes Status (mIF)

Fixed Effects	Estimate	SE	P-Value *(p)*	Sign.
Intercept	-1.889	0.877	0.031	*
Area of Islets	-0.446	0.426	0.295	
Number of Islets	0.085	0.288	0.768	
Perilipin 1 Intensity	0.408	0.265	0.124	
Tubulin beta 3 Intensity in Islets	-0.328	0.310	0.168	
Sex	0.709	0.554	0.201	
Age	0.832	0.330	0.012	*
BMI	0.593	0.263	0.024	*
Malignant Tumor	0.672	0.616	0.276	
Chronic Pancreatitis	1.915	0.967	0.048	*

Random Effects	Type	Variance	STD
Cohort	Intercept	0.754	0.868
Observations	100		
Groups	{Cohort: 2}		
Log-likelihood	-51.628		
AIC	125.256		

Z-Test (two-sided) Sign. Codes   * $p<0.05$, ** $p<0.01$, *** $p<0.001$

b. MLM Regression for HOMA2B (mIF)

Fixed Effects	Estimate	SE	P-Value *(p)*	Sign.
Intercept	0.306	0.173	0.080	
Area of Islets	-0.207	0.094	0.030	*
Number of Islets	0.280	0.103	0.008	**
Perilipin 1 Intensity	0.032	0.085	0.706	
Tubulin beta 3 Intensity in Islets	0.183	0.093	0.053	
Sex	-0.149	0.170	0.383	
Age	-0.154	0.088	0.085	
BMI	0.243	0.090	0.008	**
Malignant Tumor	0.076	0.187	0.685	
Chronic Pancreatitis	0.314	0.307	0.309	
Insulin Therapy	-1.435	0.244	3.06E-42	***

Random Effects	Type	Variance	STD
Cohort	Intercept	0	0
Observations	91		
Groups	{Cohort: 2}		
Log-likelihood	-105.898		
AIC	237.797		

Z-Test (two-sided) Sign. Codes   * $p<0.05$, ** $p<0.01$, *** $p<0.001$

Suppl. Table 3: Regression results of the generalized mixed linear model analysis for (a.) diabetes status and (b.) HOMA2B levels based on mIF stainings (*n=100*).
