# Supplementary material for: Explainable AI-based analysis of human pancreas sections identifies traits of type 2 diabetes
Source: Nat Commun. 2026 Feb 9;17:1558. doi: 10.1038/s41467-026-69295-2 (PMC12894717; doi:10.1038/s41467-026-69295-2)
Supplement: Supplementary file 2 — Reporting Summary [file 41467_2026_69295_MOESM2_ESM.pdf]

nature portfolio

Corresponding author(s): Robert Wagner

Last updated by author(s): 2025.12.04

Reporting Summary

Nature Portfolio wishes to improve the reproducibility of the work that we publish. This form provides structure for consistency and transparency in reporting. For further information on Nature Portfolio policies, see our Editorial Policies and the Editorial Policy Checklist.

Statistics

For all statistical analyses, confirm that the following items are present in the figure legend, table legend, main text, or Methods section.

n/a	Confirmed	
☐	☒	The exact sample size (*n*) for each experimental group/condition, given as a discrete number and unit of measurement
☐	☒	A statement on whether measurements were taken from distinct samples or whether the same sample was measured repeatedly
☐	☒	The statistical test(s) used AND whether they are one- or two-sided<br>*Only common tests should be described solely by name; describe more complex techniques in the Methods section.*
☐	☒	A description of all covariates tested
☐	☒	A description of any assumptions or corrections, such as tests of normality and adjustment for multiple comparisons
☐	☒	A full description of the statistical parameters including central tendency (e.g. means) or other basic estimates (e.g. regression coefficient) AND variation (e.g. standard deviation) or associated estimates of uncertainty (e.g. confidence intervals)
☐	☒	For null hypothesis testing, the test statistic (e.g. *F*, *t*, *r*) with confidence intervals, effect sizes, degrees of freedom and *P* value noted<br>*Give P values as exact values whenever suitable.*
☒	☐	For Bayesian analysis, information on the choice of priors and Markov chain Monte Carlo settings
☐	☒	For hierarchical and complex designs, identification of the appropriate level for tests and full reporting of outcomes
☒	☐	Estimates of effect sizes (e.g. Cohen's *d*, Pearson's *r*), indicating how they were calculated

Our web collection on statistics for biologists contains articles on many of the points above.

Software and code

Policy information about availability of computer code

Data collection	WSI acquisition was performed with i) i) a Hamamatsu NanoZoomer 2.0-HT using 20x magnification and NDP.scan 2.5 software for bright-field images and ii) Zeiss AxioScan.Z1 equipped with ZEN 3.10 at at 20x magnification for immunofluorescence images.<br>Data preprocessing was carried out using the python packages numpy (1.26.3), pandas (2.1.4), czifile (2019.7.2) and mzarr (0.0.7).
Data analysis	All code for data preprocessing as as well as as data analysis is custom written and is open available here: https://github.com/MIC-DKFZ/diabetes-xai

For manuscripts utilizing custom algorithms or software that are central to the research but not yet described in published literature, software must be made available to editors and reviewers. We strongly encourage code deposition in a community repository (e.g. GitHub). See the Nature Portfolio guidelines for submitting code & software for further information.

Data

Policy information about availability of data

All manuscripts must include a data availability statement. This statement should provide the following information, where applicable:

- Accession codes, unique identifiers, or web links for publicly available datasets
- A description of any restrictions on data availability
- For clinical datasets or third party data, please ensure that the statement adheres to our policy

The data is is publicly available at: https://pub.apps.dzd-ev.org/s/QiCePJeymPDZ4XY . The Password is "T2DIA6344!".

Research involving human participants, their data, or biological material

Policy information about studies with human participants or human data. See also policy information about sex, gender (identity/presentation), and sexual orientation and race, ethnicity and racism.

Reporting on sex and gender	The manuscript includes no gender-based analysis. Albeit, the sex distribution of the full patient cohort is reported and sex is included as control variable (e.g. for diabetes status or insulin secretion).
Reporting on race, ethnicity, or other socially relevant groupings	No social categorizations were used for the analysis presented in this manuscript.
Population characteristics	Cohort characteristics are as follows:<br>Tübingen cohort comprised 50 patients (38% females) aged (years) 63.2±12.97, with a BMI (kg/m2) of 26.33 ± 5.8. 18% of the patients were diagnosed with type 2 diabetes (disease duration of 2.2 ± 6.5 years), 58% had a malignant disease and 6% chronic pancreatitis. 12% of the patients received metformin and 2% had a sulfonylurea treatment.<br>Dresden cohort comprised 50 patients (52% females) aged (years) 61.8 ± 14.35, with a BMI (kg/m2) of 24.30 ± 4.1. 52% of the patients were diagnosed with type 2 diabetes (disease duration of 5.7 ± 8.8 years), 46% had a malignant disease and 22% chronic pancreatitis. 11% of the patients received metformin, 4% had a sulfonylurea treatment, 6% received a treatment with GLP1 analog and 2% a SGLT2 inhibitor.
Recruitment	Patients undergoing pancreatic surgery for different indications at two academic sites of the German Center for Diabetes Research network: the University Hospital Tübingen and the University Hospital Dresden.
Ethics oversight	The study was approved by the Ethical Committees of the Technische Universität Dresden (Reference EK 151062008) and Eberhard Karls Universität Tübingen (Reference 539 697/2011BO1).

Note that full information on the approval of the study protocol must also be provided in the manuscript.

Field-specific reporting

Please select the one below that is the best fit for your research. If you are not sure, read the appropriate sections before making your selection.

☒ Life sciences ☐ Behavioural & social sciences ☐ Ecological, evolutionary & environmental sciences

For a reference copy of the document with all sections, see nature.com/documents/nr-reporting-summary-flat.pdf

Life sciences study design

All studies must disclose on these points even when the disclosure is negative.

Sample size	Number and distribution of recruited patients: In total, 100 metabolically phenotyped pancreatectomized living donors, 50 per treatment site (a) University Hospital Dresden and (b) University Hospital Tübingen, were randomly selected from all available patients matching the recruitment criteria (Dresden: 348; Tübingen: 179) with respect to their diabetic status and sex. Composition of the Dresden cohort: 15 non-diabetic (ND) females (♀); 9 ND males (♂); 11 type 2 diabetic (T2D) ♀; 15 T2D ♂. Composition of the Tübingen cohort: 15 ND ♀; 26 ND ♂; 4 T2D ♀; 5 T2D ♂.<br>For each patient, 10 consecutive sections of the same pancreas fragment were stained and imaged either (i) chromogenically (6 sections: one per marker (A) insulin, (B) glucagon, (C) somatostatin, (D) PECAM1, (E) perilipin 1, and (F) tubulin beta 3), or (ii) fluorescently (4 sections: two per Stainingset 1 (glucagon, somatostatin, and tubulin beta 3) and Stainingset 2 (insulin, perilipin 1, and PECAM1)).
Data exclusions	Patients were excluded from the selection due to pancreatic-disease related diabetes diagnosed within a year prior surgery (Dresden: 114 from total 462; Tübingen: 25 from total 204).
Replication	Data was acquired once per patient (clinical data) or pancreatic section. In total, 100 data sets of clinical data, 600 chromogenic whole slide images, and 100 fluorescence whole slide images were duplicates per two staining sets (in total 400).
Randomization	In total, 100 metabolically phenotyped pancreatectomized living donors, 50 per treatment site (a) University Hospital Dresden and (b) University Hospital Tübingen, were randomly selected from all available patients matching the recruitment criteria (Dresden: 348; Tübingen: 179) with respect to their diabetic status and sex.
Blinding	Patients were split into a training set (n=75) and a test set (n=25), with the test set blinded for trained models and used only for the final model evaluation.

Reporting for specific materials, systems and methods

We require information from authors about some types of materials, experimental systems and methods used in many studies. Here, indicate whether each material, system or method listed is relevant to your study. If you are not sure if a list item applies to your research, read the appropriate section before selecting a response.

Materials & experimental systems

n/a	Involved in the study	
☐	☒	Antibodies
☒	☐	Eukaryotic cell lines
☒	☐	Palaeontology and archaeology
☒	☐	Animals and other organisms
☐	☒	Clinical data
☒	☐	Dual use research of concern
☒	☐	Plants

Methods

n/a	Involved in the study	
☒	☐	ChIP-seq
☒	☐	Flow cytometry
☒	☐	MRI-based neuroimaging

Antibodies

Antibodies used	For chromogenic staining following antibodies were used: insulin (Dako, #A0564), glucagon (Santa Cruz, #sc13091), somatostatin (Invitrogen, #14-9751-80), CD31 (Dako, #M0823), perilipin 1 (Progen, #651156) and tubulin beta 3 (R&D Systems, #MAB1195). The secondary horseradish peroxidase-<br>linked antibody was part of of the Opti-View DAB IHC detection kit (Roche Ventana, #06396500001). The antibody cocktail for fluorescent Stainingset 1 included: anti-<br>glucagon (Abcam plc. #Ab10988), anti-tubulin beta 3 (R&D Systems, MAB1195), and anti-somatostatin con. AF750 (Novus Biologicals, #NBP2-99309 AF750), mouse IgG1 con. AF555 (Invitrogen, #A-21127) and mouse IgG2a con. AF647 (Invitrogen, #A21241). The antibody cocktail for Stainingset 2 included anti-perilipin 1/PLIN1 (Progen, #690156), anti- PECAM1/CD31 (Abcam, #ab134168), insulin con. AF488 (Invitrogen, #53-9769-82), mouse IgG1 con. AF555 (Invitrogen, #A21127) and rabbit IgG con. AF750 (Invitrogen, #A21039).
Validation	Validation statements on on the manufacturer's websites and previous publications using respective antibodies were considered. The antibodies were tested for appropriate dilutions.

Clinical data

Policy information about clinical studies

All manuscripts should comply with the ICMJE guidelines for publication of clinical research and a completed CONSORT checklist must be included with all submissions.

Clinical trial registration	*Provide the trial registration number from ClinicalTrials.gov or an equivalent agency.*
Study protocol	*Note where the full trial protocol can be accessed OR if not available, explain why.*
Data collection	*Describe the settings and locales of data collection, noting the time periods of recruitment and data collection.*
Outcomes	*Describe how you pre-defined primary and secondary outcome measures and how you assessed these measures.*

Plants

Seed stocks	*Report on the source of all seed stocks or other plant material used. If applicable, state the seed stock centre and catalogue number. If plant specimens were collected from the field, describe the collection location, date and sampling procedures.*
Novel plant genotypes	*Describe the methods by which all novel plant genotypes were produced. This includes those generated by transgenic approaches, gene editing, chemical/radiation-based mutagenesis and hybridization. For transgenic lines, describe the transformation method, the number of independent lines analyzed and the generation upon which experiments were performed. For gene-edited lines, describe the editor used, the endogenous sequence targeted for editing, the targeting guide RNA sequence (if applicable) and how the editor was applied.*
Authentication	*Describe any authentication procedures for each seed stock used or novel genotype generated. Describe any experiments used to assess the effect of a mutation and, where applicable, how potential secondary effects (e.g. second site T-DNA insertions, mosiacism, off-target gene editing) were examined.*
